# Effects of Modified Metakaolin Using Nano-Silica on the Mechanical Properties and Durability of Concrete

**DOI:** 10.3390/ma12142291

**Published:** 2019-07-17

**Authors:** Nasir Shafiq, Rabinder Kumar, Muhammad Zahid, Rana Faisal Tufail

**Affiliations:** 1 Department of Civil and Environmental Engineering, Universiti Teknologi PETRONAS, Bandar Seri Iskandar 32610, Perak, Malaysia; 2 Department of Civil Engineering, GIK Institute of Engineering Sciences and Technology, Topi 23460 KPK, Pakistan; 3 College of Architecture and Environment, Sichuan University, Chengdu 610065, China

**Keywords:** response surface method, concrete durability, nano-silica, concrete, mercury porosimetry

## Abstract

This paper discussed the effects of modified metakaolin (MK) with nano-silica (NS) on the mechanical properties and durability of concrete. In the first phase, trial mixes of concrete were prepared for achieving the desired value of the 28 days compressive strength, and the charge passed in rapid chloride permeability test (RCPT). In the second phase, statistical analysis was performed on the experimental results using the response surface method (RSM). The RSM was applied for optimizing the mix proportions for the required performance by exploiting the relationship between the mix characteristics and the corresponding test results. A blend of 10% MK + 1% NS as part of cement replacement exhibited the highest mechanical properties and durability characteristics of concrete; concrete mix showed that the 28-days compressive strength (CS) was 103 MPa, which was 15% greater than the CS of the control mix without MK or NS. The same mix showed more than 40% higher flexural and split-tensile strength than the control mix; also it resulted in a reduction of 73% in the rapid chloride permeability value. ANOVA technique was used for optimizing the nano-silica and metakaolin content for achieving maximum compressive strength and minimum RCPT value. Statistical analysis using ANOVA technique showed that the maximum compressive strength and lowest RCPT value could be achieved with a blend of 10% MK and 1.55% NS.

## 1. Introduction 

Concrete is widely used construction material for more than a century for the rapid development of the built environment all over the world. New forms of concrete have been evolved through many advances via dedicated research and development over many decades. For example, in the first evolution cycle, researchers focused on increasing the compressive strength, and now the focus has changed to improve the functional characteristics and performance of concrete. For this current evolution in concrete technology, advances in the development and application of nano-materials have played a significant role. There are multiple reasons for the need of continuous evolution of concrete technology; one of them is the concerns on the greenhouse gas emission and depletion of natural resources for the mass scale production of cement; which is the primary constituent material of concrete. It is understood that the concrete structures can withstand well the harsh environmental conditions (water logging, salinity, chloride exposure, etc.) that may result in structural damage [1]. Corrosion of steel reinforced is considered one of the principal causes of poor durability, the ingress of chloride ions in concrete from the ambient conditions is the primary source of corrosion. The effects of steel reinforcement corrosion in concrete include the loss of strength, reduced durability, and dull aesthetic of the structural system [2]. Over the last few years, structural damages due to steel reinforcement corrosion have increased to a massive scale that has tremendously increased the maintenance and repair cost of the structures [3,4]. To resolve the issues and concerns (Green House Gas (GHG) emission, natural resource depletion, and poor performance) raised with the increasing use of concrete, many alternative solutions are found through research and development efforts [5]. One of the efforts is introducing the value-added cement replacement materials (CRM) in the new recipe of concrete. Some of the value-added materials, i.e., fly ash (FA), silica fume (SF), and ground granulated blast-furnace slag (GGBS) have been widely used that improve the service life and reduce the maintenance cost of the structure [6]. The benefits of such materials were obtained because they refined the pore structure of concrete by blocking the pore connectivity, which results in lower permeability and higher strength [7].

Metakaolin (MK) is another kind of CRM, which is included in the CRM group two decades ago, because it offered some added benefits over the use of the other types of CRM [8,9]. Metakaolin is obtained by incinerating kaolin clay at higher temperatures within the range of 600 °C to 800 °C; it usually contains highly amorphous silica and alumina content [10]. Dinkar et al. [11] found 10% MK content as the optimum replacement of cement for achieving the high compressive strength, which also reported in the other studies [12,13]. The other benefits of 10% MK in concrete are obtained as the reduced water permeability, absorption, and chloride permeability. Some research studies found that more than 10% of MK content caused a decrease in the compressive strength, which may be due to the clinker dilution effect [13]. However, Wild et al. [14] reported that a dosage of 20% replacement of MK was observed effective as it facilitated to gain in the strength at a 0.45 w/c ratio. Kim et al. [15] conducted a comprehensive investigation by using five replacement levels (0% to 20%) of MK and SF in combination with 25% of fly ash content; this study also found 10% MK content as the optimum replacement level. Mardani et al. [16] compared the results of fly ash, metakaolin, and silica fume; the researchers used 10% replacement level for all three materials, it was found that metakaolin and silica fume performed well to improve the sulfate resistance of concrete.

In the continued efforts for advancement in concrete technology, researchers anticipated that the rapid developments in the nano-science might be useful for advancing concrete to the next generation development that is focused on achieving the desired functional characteristics and sustainability objectives. In this regards, some research studies investigated the effects of nano-silica on the performance of concrete and observed a significant enhancement in the compressive strength than the controlled mix made of 100% cement [17]. Nazri and Rihai [17] different nano-silica content from 0% to 2% and concluded that 2% of NS content resulted in the highest compressive strength of concrete. In contrary to Riahi and Nazari findings, some other researchers observed no or minimal effects of NS on the compressive strength of concrete [18]. Jalal et al. [19] blended 2% NS with silica fume and fly ash for exploring the triple blended cement effects on the mechanical and thermal properties, transport mechanism, and microstructure of concrete. Apart from the effects of nano-silica on the compressive strength of concrete, research investigations also studied the effects of NS on the durability and long-term performance of concrete. Said et al. [20] prepared concrete samples containing a dosage of 3% and 6% NS for measuring the chloride ingress and found a reduction of 33% and 22% respectively in the depth of penetration also the samples showed quite low value of total the charge passed during rapid chloride penetration test (RCPT), which is an indication of high durability. According to the fundamental principles of concrete technology, porosity, pore size, and pore-connectivity are the controlling factors for achieving higher compressive strength and long-term durability [21]. In an experimental investigation, Ghafari et al. [22] observed a reduction in the total porosity from 24.5% to 32.1% with the addition of 1% to 4% nano-silica content. Similarly, when such concrete samples were tested for water absorption, they showed a decrease in the penetration depth from 8% to 33%. 

The principal aim of this research study was to investigate the effects of modified metakaolin using nano-silica as partial replacement of cement on the mechanical properties and durability characteristics of concrete. For this purpose, a number of trial mix proportions were prepared, and samples were cast, cured, and tested for compressive, split tensile, and flexural strength tests. Durability characteristics were investigated using the rapid chloride permeability test, and porosity using mercury intrusion porosity tests. Experimental results of all trial concrete mixes were statistically analyzed using ANOVA technique for finding an optimum combination of metakaolin and nano-silica for achieving the highest desired compressive strength and the long-term durability.

## 2. Materials

Materials used in this research were Type-I ordinary Portland cement (OPC) complying the requirements of ASTM C150, metakaolin (MK) and nano-silica (NS) were supplied by a local vendor in Malaysia. Nano-silica was comprising of 99.8% of SiO_2_ and the median grain diameter of 20 nm. The chemical composition of cement and metakaolin are shown in Table 1, whereas, Table 2 shows the physical and chemical composition of nano-silica. Coarse aggregate (crushed granite) with a maximum size of 14 mm was obtained from a local quarry in the state of Perak, Malaysia and mining sand was used as a fine aggregate that was supplied by a local supplier. An aqueous solution of modified poly-carboxylates superplasticizer (SP) was used to achieve the desired slump (between 100 mm to 130 mm) of fresh concrete, because the water-to-binder ratio (w/b) was kept constant at 0.35 for all concrete mixes. 

### 2.1. Mix Design Optimization Using Response Surface Method (RSM)

Response surface methodology (RSM) is a combination of statistical and mathematical techniques is used for analyzing a function where several independent variables influence a dependent variable called a response. The principal objective of applying RSM is to achieve an optimum outcome of the desired response. The RSM is a four-step process: (1) The design of experiments; (2) conducting an experiment for obtaining the desired response; (3) modeling of factors and response, based on experimental results; and finally, (4) optimization and validation of the model [23]. The most commonly used design method in finding the functional relationship between the response and the factors using RSM is the central composite design (CCD) [24]. 

It is discussed in the literature that using high value-added materials (such as nano-silica) in the modern concrete mix proportion is to expect a high-performance of structure throughout the service life. The term high performance is referred to achieve the highest response (mechanical strength and durability) with the optimum mix proportions. For this study, the desired responses were; compressive strength, porosity, rapid chloride permeability, and chloride penetration depth, whereas, metakaolin and nano-silica were considered as the influencing factors [25]. Level of variations of the controlling factors (metakaolin and nano-silica content) was adopted by referring to existing literature [26,27,28] and performing a trial mixing and testing program. The considered factors (metakaolin and NS content) were varied in three levels, i.e., metakaolin (0%, 5%, and 10%) and nano-silica (0%, 1% and 2%). The maximum level of 10% for MK content and 2% of NS content was set using trial tests study, which showed adverse results when such proportion was exceeded. Existing literature also indicated a similar threshold for using MK and NS in concrete [16,28,29]. For this study, Design Expert Software was used for the experiment design, modeling, statistical analysis, and optimization of the variables and analysis of variance (ANOVA) technique was employed to investigate the interaction between variables.

### 2.2. Trial Mix Proportions for Achieving Desired Compressive Strength

In this study, initial experiments were designed using face-centered central composite design (FCCCD) approach using the Design-Expert Software. In the trial mixing and testing program; nine mixes were prepared with constant water to binder ratio (w/b) of 0.35, and the total binder content (cement + MK + NS) was set as 500 Kg/m^3^, details of all mixes are given in Table 3. The compressive strength of concrete was considered as one of the responses; the desired value was set at 90 ± 5 MPa. The desired strength was achieved using a trial mix proportioning and testing approach, because there is no available mix-design procedure for high compressive strength. The target strength was achieved by varying MK content from 0% to 10% and NS content from 0% to 2%. In case the desired slump was not achieved using constant w/b of 0.35, an appropriate amount of superplasticizer was added to achieve the target value.

## 3. Mixing, Molding, and Testing of Specimen

A 100 kg laboratory mixer was used for preparing concrete mixes; the mixing operation complied the requirements of BS EN 206-1 and complementary British standard BS 8500-2. Nano-silica was directly added to concrete constituents. However, special care was taken that it should be uniformly dispersed throughout the mix. Immediately after mixing of all concrete mixes, slump test was performed according to BS 1881: Part 102 to verify the achievement of the desired slump between 100 mm to 130 mm. 

Hardened concrete was tested for determining the mechanical properties and durability characteristics. Cube compressive strength, splitting tensile strength, and the flexural strength was determined for investigating the mechanical properties of concrete. For compressive strength, 100 mm cubes were tested at the age of 3, 7, 28, and 90 days according to the specifications of BS-1881-116 and BS-EN 12390-3. The splitting tensile strength test was performed at the age of 28 days on 100 × 100 × 100 mm^3^ diameter and 200 mm long cylindrical samples complying with the requirements of BS-EN 12390-6. Similarly, flexural strength was performed at the age of 28 days on 500 × 500 × 100 mm^3^ samples, according to BS-EN 12390-5. 

In the second phase of the hardened concrete testing program, durability characteristics were determined using chloride migration and pore structure analysis tests. For chloride migration, rapid chloride permeability test and immersion test for measuring chloride penetration depth were performed. Whereas, pore structure investigation was done using mercury intrusion porosimetry (MIP) that measured the total porosity and median pore diameter of the samples. The rapid chloride permeability test (RCPT) was performed at the age of 28 days according to AASHTO T277 or ASTM C1202, 100 mm diameter and 50 mm thick vacuum saturated concrete discs were used. The testing rig is composed of two cells; the first cell is filled with 3% NaCl solution, whereas, the second cell is filled with 0.3 M NaOH solution, the sealed concrete disc is placed in between the two cells. The RCPT is performed at 60 volts for 6 hours, during the test the current was measured at an interval of 3 minutes; at the end of the test time versus current plot was used to determine the total charge passed, which is represented in coulombs. According to ASTM, C1202-17 total charge passed is an indication of chloride permeability of concrete. Immersion test is a static test, which takes longer time if concrete pore structure is very tight. In performing the tests, concrete discs were immersed in a 3% NaCl solution until the age of testing. At the end of immersion, the discs were split into two halves, and AgNO_3_ was sprayed on the split half to distinguish the depth of penetration, the salt solution usually turn to silver color when exposed to AgNO_3_ solution. Porosity, pore size, and pore connectivity is an essential property of controlling the mechanical strength and durability of concrete. For this study, mercury intrusion porosimetry (MIP) was used to investigate the pores characteristic of concrete. The test was done at the age of 28 days; approximately 10 mm size mortar cores were used for testing. Samples were tested at the oven-dry condition; for that reason, samples were dried at 100 °C for approximately 48 hours [30].

Field Emission Scanning Electron Microscopy (FESEM) analysis was performed to investigate the effects of nano-silica on the microstructural properties of mortar reaction product; the analysis also determined the characteristics of the interfacial transitional zone (ITZ) of the mortar samples cured for 28 days. For this study, FESEM model Zeiss Supra 55 VP instrument was used. The samples were cut using a diamond cutter, which was supposed to be conducive for full observation. Therefore, the samples were coated accordingly prior to FESEM analysis; they were coated with gold atoms in a sputter coater. The coated samples were then placed in the vacuum chamber inside the FESEM testing equipment.

## 4. Results and Discussion

### 4.1. Investigating the Effects on the Mechanical Properties

In the first phase, the effects of nano-silica and metakaolin were investigated on the mechanical properties (compressive, splitting tensile, and flexural strength). The experimental results are discussed in the following sections.

#### 4.1.1. Effect of NS and MK on Compressive Strength

**Figure 1:**
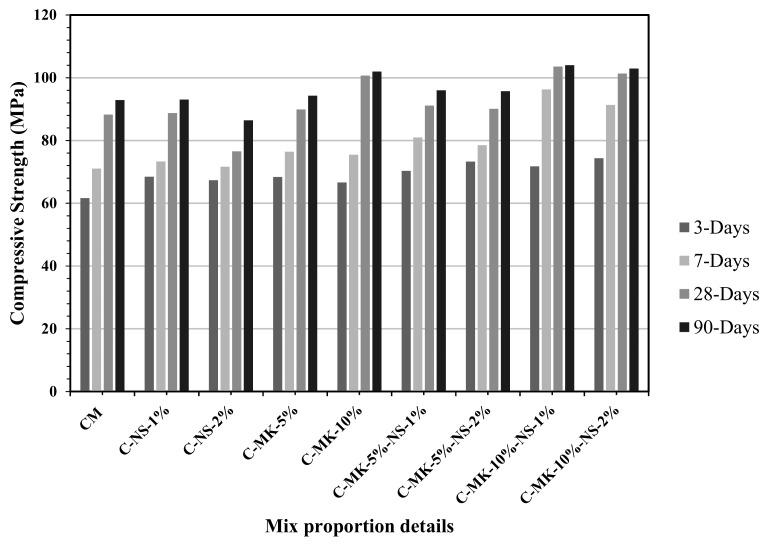
The effects various combinations of MK and NS on the compressive strength of concrete determined at a different age.

Figure 1 shows the effects of different combinations of nano-silica and metakaolin content on the compressive strength determined at the age of 3, 7, 28, and 90 days. It is observed that if only nano-silica is added to the mixture, it did not appreciably affect the compressive strength of concrete. When a dosage of 1% NS added, it resulted in an increase of less than 5% in the compressive strength determined at all ages. Whereas, using 2%, NS caused a slight reduction (less than 3%) in the compressive strength at the age of 28 and 90 days. On the other end, with 10% MK, about a 22% increase in the compressive strength was obtained at the age of 28 and 90 days. Therefore, it was anticipated that a combination of NS and MK would result in a positive manner on the performance of concrete. A combination of 1% NS and 10% MK content showed the highest compressive strength all ages as compared to all other concrete mixes. This combination exhibited 103.60 MPa and 104.02 MPa of compressive strength at the age of 28 days and 90 days, which was 15.2% and 11.9% higher than the relevant control mix. A possible reason for the reduction in compressive strength with the addition of more than 1% NS could be the agglomeration of nanoparticles. In principle, nanoparticles have a higher surface area and high surface energy, which causes particles to get agglomerated and form larger pores inside the concrete [31]. Whereas, regarding the combination of NS and MK, the increase in the compressive strength would be due to the filler effect, and the pozzolanic reaction happened using both materials. The fine particles of metakaolin and nano-silica tend to fill the voids and refined the pore structure of concrete. Also, the pozzolanic reaction of nano silica can consume portlandite, which not only reduces the crystal size, but also caused the hydration products to become more homogenous. Due to such facts, it has a noticeable effect at the interfacial transition zone (ITZ) which creates a better bond between aggregates and cement paste, hence, caused an improvement in the mechanical performances of concrete [32,33].

#### 4.1.2. Effects of NS and MK on Split Tensile and Flexural Strength

**Figure 2:**
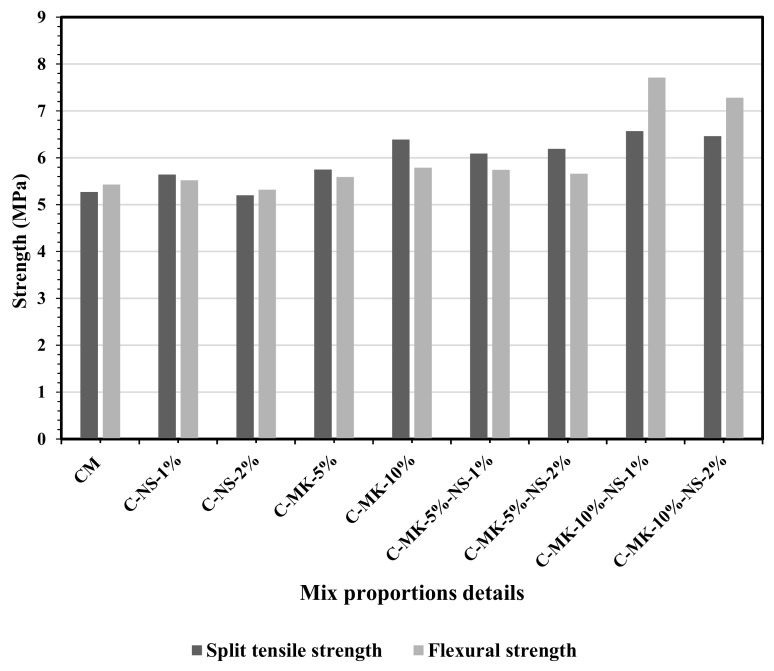
The effects various combinations of MK + NS on the flexural and split tensile strength of concrete determined at the age of 28 days.

Figure 2 shows the effects of different combinations of NS and MK on the split tensile and flexural strength of different concrete mixes. If the only dosage of NS was added, it has exhibited a similar pattern of achieving the splitting tensile and flexural strength of concrete as it was observed with the compressive strength test. 1% NS content caused about 7% increment in the split tensile strength as compared to that of the control mix. The reason for increasing the concrete tensile strength is probably due to the strong bond between paste and aggregates, which was due to highly refined particles of NS [30]. Different combinations NS and MK have shown higher splitting tensile strength than all other mixes. Concrete containing 5% MK, and 1% and 2% NS showed 15.5% and 17.5% increase in splitting tensile strength at the age of 28 days. Whereas, the optimum combination of 1% NS with 10% MK showed an increment of 19.78% in splitting tensile strength.

Combination of MK and NS showed a reasonable increase in the flexural strength of concrete. Combination of 10% MK and 1% NS resulted in 43% and 32% increase in the flexural strength respectively than that was obtained for the control mix and mix (C-MK-10%). However, the addition of NS more than 1% showed a slight decrease in the flexural strength. A similar trend was observed for the tensile strength of NS/MK concrete. It can be concluded that a combination of 10% MK and 1% NS caused a significant improvement in flexural strength, as well as tensile strength, which can be termed as an optimum. Kim et al. [29] discussed that 10% content of MK is accepted as the optimum level, considering economic efficiency. A similar effect of metakaolin has been observed by Wild et al. [14]. The contribution of MK to make the higher strength of concrete is based on three main factors. These are the filler effect (which is instant), the acceleration of Portland cement hydration (occurs within the first 24 hours), and the pozzolanic reaction [29].

### 4.2. Effects of MK and NS on the Durability Characteristics

In the next phase of the study, the effects of nano-silica and metakaolin were studies on the durability characteristics of concrete. There were various tests conducted that can indicate the level of durability of concrete. In the following sections, results of various durability tests are discussed.

#### 4.2.1. Water Absorption

**Figure 3:**
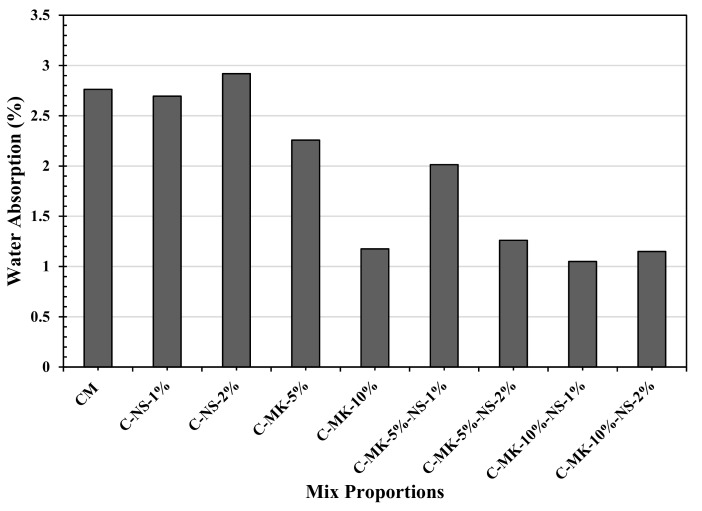
Effects of the combination of MK and NS on the water absorption capacity of concrete.

Water absorption test gives an indirect indication of concrete durability; the faster and deeper penetration of water will be an indication of inadequate durability. Figure 3 shows the results of the water absorption test conducted at the age of 28 days. It is observed that the concrete samples made of a combination of nano-silica and metakaolin resulted in a low penetration depth of water, which is an indication of higher durability. The concrete containing 10% MK showed a 57% reduction in the penetration deep as compared to that obtained for the control mix, 10% MK concrete also resulted in 47% less penetration depth the concrete made of 5% MK + 1% NS. The results obtained in this study are concurring with the results of Khatib and Clay [34], and Ramezanianpour et al. [35] the reason of reduction of penetration with 10% MK could be the refinement of pore structure.

Similarly, the combination of MK and NS also caused a significant reduction in water penetration depth. An addition of 1% and 2% NS with 5–10% replacement of MK has shown 27% and 54% reduction in penetration depth respective as compared to the penetration depth obtained in the control mix. Results showed that the addition of nano silica has higher compatibility with 10% of metakaolin on the reduction of water absorption in concrete.

#### 4.2.2. Rapid Chloride Permeability Test

**Figure 4:**
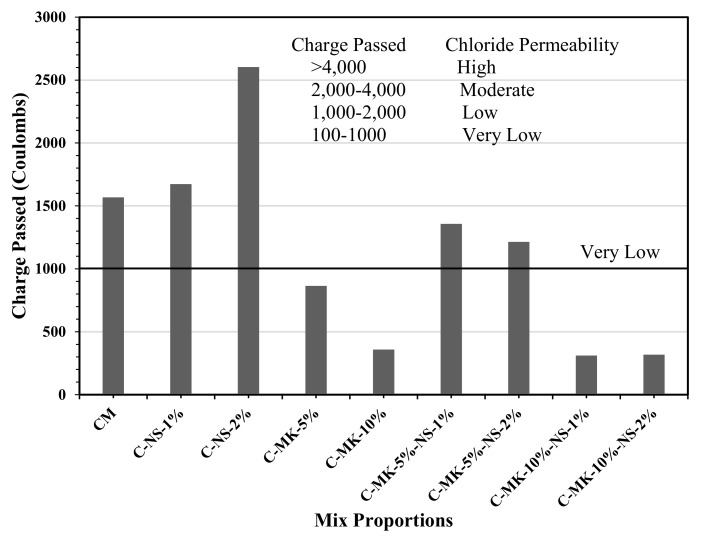
Rapid Chloride Permeability (Coulombs) for all concrete mixes.

The rapid chloride permeability (RCP) test for all concrete mixes was carried out at the age of 28 days; Figure 4 shows the results of RCP as charge passed, Coulombs. Concrete mixes made of the combination of MK and NS showed a quite low value of charge passed (Coulombs); according to ASTM C1202, if RCP is obtained less than 1000 Coulombs for any concrete mix, it means that the concrete will achieve long term durability during the life of the structure. The concrete mixes made of 5% and 10% MK resulted in a value of charge passed as 864 and 358 Coulombs respectively. The concrete mix containing 10% MK achieved a 77% reduction in the RCP value than that was determined for the control mix; similar trends in results were observed in the previous studies [29,35]. In a recent publication, Alaily and Hassan [36] also reported that the RCP value of concrete is reduced with an increase in the MK content in concrete, which was caused due to pore filling effects of MK and the pozzolanic activity that refined the pore-system and densified the concrete matrix. Therefore, it is generalized that the addition of MK enhances the chloride binding capacity of concrete. Hence, the concentration of free chloride ion in the pore water is reduced; the higher concentration of chloride ions in the pore water increases the chances of corrosion of embedded steel reinforcement [37]. When only 1% and 2% nano-silica was added in concrete; the RCP of concrete was increased as compared to that obtained for concrete with 5% and 10% MK content. The increase in RCP of NS concrete may be caused due to the reduction in strength, because of nano-silica cause agglomeration of nanoparticles due to their higher surface area and high surface energy. The agglomeration of nanoparticles resulted in larger size pores. However, a combination of MK and NS in concrete showed a significant improvement in the RCP. When 1% and 2% NS content was combined with 10% MK, it showed a remarkable reduction in the total charge passed in concrete; the values were obtained as 311 Coulombs and 318 Coulombs respectively. One of the reasons could be the filler effects due to fine particles of MK and NS that also resulted in higher compressive strength.

#### 4.2.3. Chloride Penetration Depth

Measurement of chloride penetration depth in concrete is a static test that represents the real-life situation. Chloride penetration depth determines the concrete cover requirements for achieving the desired service life of the structure. The chloride penetration depth, CPD was measured after three months immersion of concrete disc in 3% salt solution. CPD of the control mix was obtained as 11.83 mm, and for 5% and 10% MK concrete, it was measured as 5.80 mm and 9.00 mm respectively, the CPD 48% and 19% lower than the control mix. As discussed above that 1% and 2% dosage of NS with 10% MK caused a significant reduction in RCPT value, which trend is also shown in the reduction of CPD, which was reduced to 71% and 74% respectively with respect to the control mix CPD. 

#### 4.2.4. Mercury Intrusion Porosimetry

This technique is preferred when assessing the porosity and pore size distribution of cement paste and concrete. The concrete pores are classified into three categories; pores in the range of 50> nm, 10–50 nm and <10 nm, are designated as “large capillary pores,” “medium capillary pores” and “gel pores” respectively. The difference in pore size leads to different effects on concrete strength and durability performance [38]. The capillary pores are termed as macropores or harmful pores. Generally, the strength and durability of concrete are affected by large capillary pores. Gel pores form a part of CSH is considered as micropores. They are inactive in water permeability and does not contribute any impact on the strength [39].

**Figure 5:**
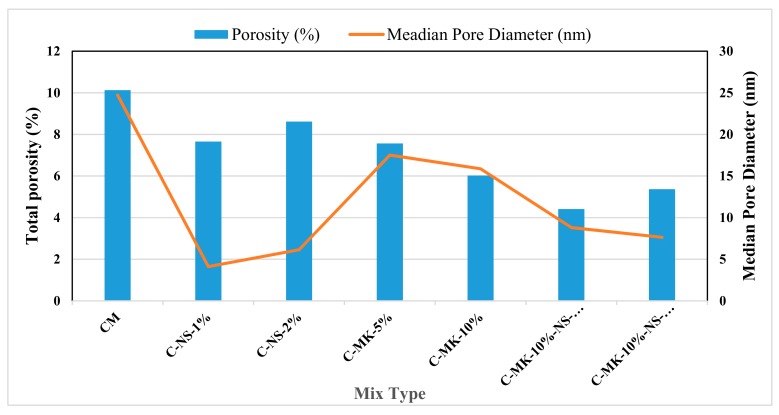
Total Porosity and Median Pore Diameter of all concrete mixes.

Figure 5 shows the median pore diameter and total porosity of different concrete, mixes are plotted. As obtained in other tests results that MK and MK+NS have resulted in better performance. For achieving such performance, concrete pore characteristic (pore size and porosity) play an essential role. Concrete mixes containing metakaolin was less porous than the control concrete; other researchers reported a similar trend of results [40,41]. Addition of 1% NS showed a significant decrease in porosity (P = 7.9%) with respect to the control mix. However, with 2% NS, porosity was obtained as 8.35% that was higher than the C-1%NS concrete, but it was about 20% lower than the porosity of the control mix. The increase in total pore volume was observed with the addition of higher percentages of NS into the concrete. Combination of MK and NS also showed a significant decrease in porosity and the size of pores, which indicates that the MK and NS have better compatibility to improve the microstate of concrete. The porosity of concrete depends on the continuity of the pores, pore size, and pore volume. Combination of MK and NS showed a reduction in porosity, and this reduction is due to two reasons. First the filler effect which improved the particle packing due to finer particles by blocking the interconnectivity of pore system, and the second is pozzolanic reactivity, due to which the particles reacted with Ca(OH)_2_ and at the same time acted as nucleation sites, leading to the better development of hydration products in such position [41].

#### 4.2.5. Field-Emission Scanning Electron Microscopy (FESEM)

**Figure 6:**
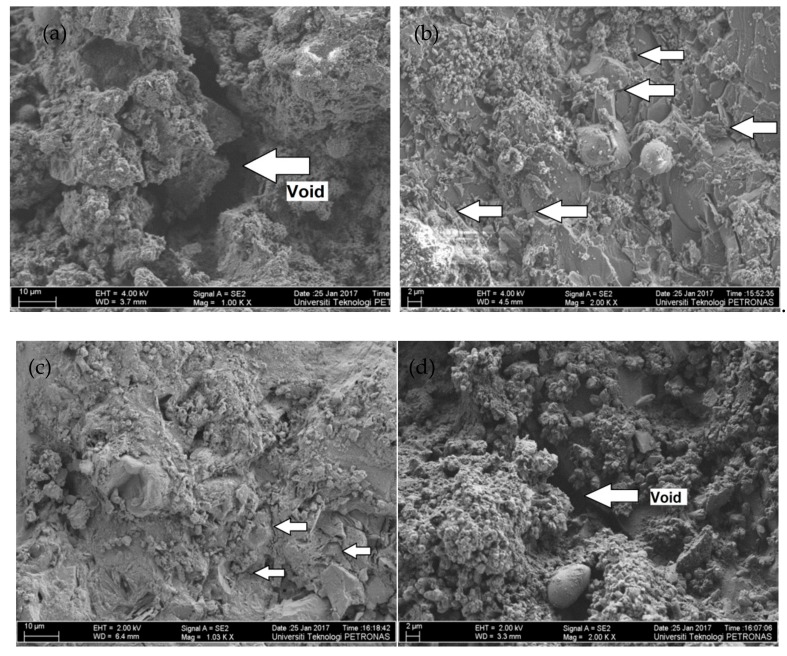
Micrographs of different concrete mix design (**a**) CM (100% Cement (Arrow pointing void), (**b**) C-MK-10% (Arrows pointing narrow gap), (**c**) C-MK-10%-NS-1% (Arrows pointing narrow gaps), (**d**) C-MK 10% and NS 2% (Arrow pointing void).

The effects of metakaolin with and without the combination with nano silica on the microstructure were investigated using FESEM. Figure 6a shows the FESEM images of the control mix (containing 100% cement), the arrow indicating void is representing interfacial transition zone of a larger width 6.84 µm, which is evident as the cement particles are coarser than the metakaolin and NS particles. Figure 6b the image of the mix containing 10% metakaolin, it can be seen that the microstructure is quite refined in this case, most of the MK particles are fully reacted, whereas, few spheres indicating the partially reacted metakaolin particles. The arrows marked as narrow gap are representing the tight ITZ, it is because of the fine particle size of MK that filled the void to much extent that is shown in Figure 6a, the gap was too narrow, therefore it was difficult to measure with this resolution. Figure 6c shows the FESEM image of a mix containing 10% MK with 1% NS; the effects are found similar to that discussed in Figure 6b. Similar findings are reported by Duan et al. [42]. Figure 6d shows the micrograph of the mix containing 10%MK with 2% NS: In this case the gap marked as void (also ITZ) looks a little wider than that observed in Figure 6a,b, and the MIP test showed about 8% higher porosity of 2%NS samples than the porosity of 1%NS sample. This showed that a part of NS in the mix remains unreactive when it exceeded 1% threshold level. Previous research studies stated that the improvement in the microstructure containing MK is due to filler effect of metakaolin and formation of secondary calcium silicate hydrates gel which bridge the gap between matrix and aggregates and makes the paste less porous and stronger [43,44]. Thus, metakaolin performed better in enhancing the microstructure of concrete. Improvement in microstructure also results in improved mechanical and durability properties. The addition of NS with MK further enhanced the microstructure; however, when the dosages exceed the threshold level, particles get agglomerated, resulting in wider ITZ, as discussed in Figure 6d.

### 4.3. Correlation of Responses

To establish statistical correlation among various responses obtained from experimental tests is a widely used approach by the researchers for the appraisal of experimental findings [35,43,45,46]. In establishing the correlations effects of the influential factors on various properties are considered. For example; the chloride migration in concrete is primarily governed by the pores characteristics and structuring, including the quality of the interfacial transition zone. Poon et al. [43] obtained a correlation between compressive strength and porosity of different concrete mixes that showed a moderate confidence level in the resulted correlation. In an experimental study, Rezaifar et al. [47] determined a correlation among various parameters, i.e., compressive strength, water absorption, and dry density. R-squared (R^2^) is a statistical measure of how close the data are to the fitted regression line. R-squared is always between 0 and 1. In general, R-squared closer to 1 indicates the effectiveness of the model. They reported a good correlation was obtained between compressive strength and dry density, whereas, not very strong correlation found between compressive strength and water absorption (R^2^ was obtained as 0.63). Ramezanianpour et al. [35] investigated concrete samples containing metakaolin for various tests, i.e., compressive strength, rapid chloride permeability, water penetration depth, and surface resistivity test. They determined an exponential correlation between different test results. A reasonably well correlation between the results of compressive strength and chloride permeability test was obtained (R^2^ was obtained as 0.814). 

**Figure 7:**
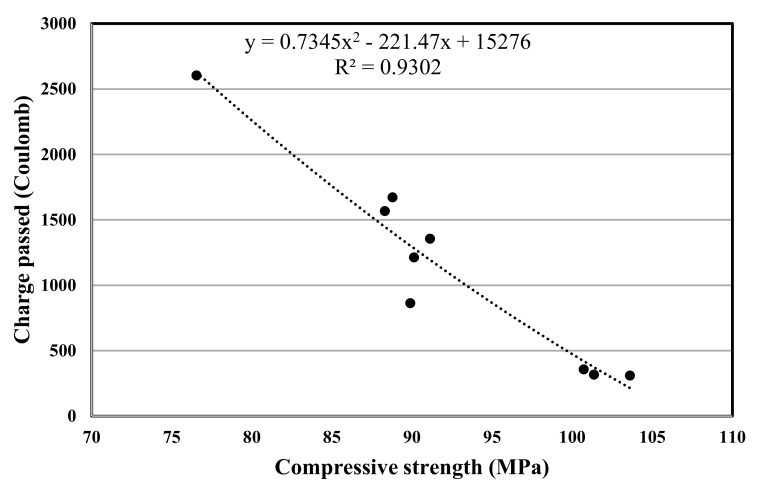
Correlation between compressive strength and the total charge passed.

**Figure 8:**
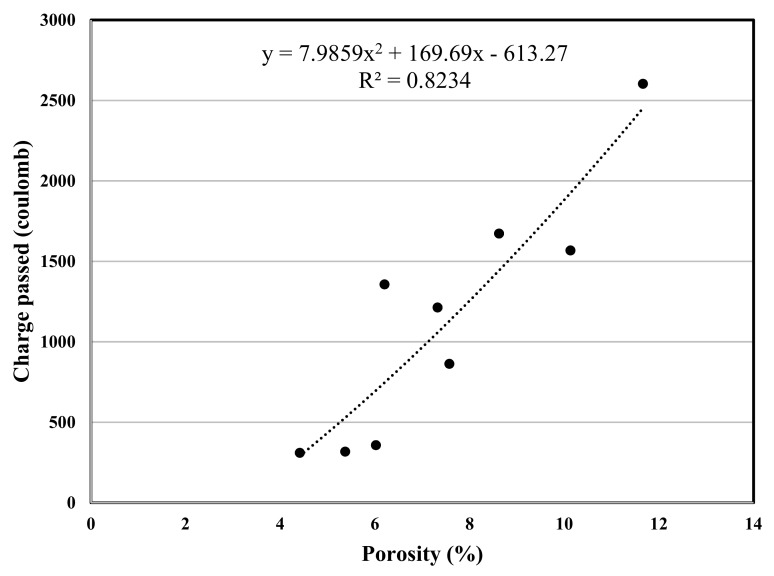
Correlation between porosity and total charge passed.

**Figure 9:**
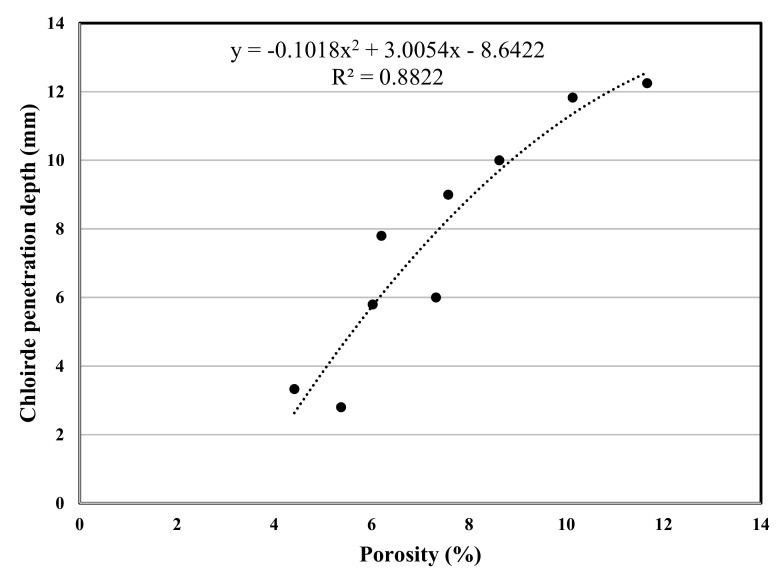
Correlation between porosity and chloride penetration depth.

**Figure 10:**
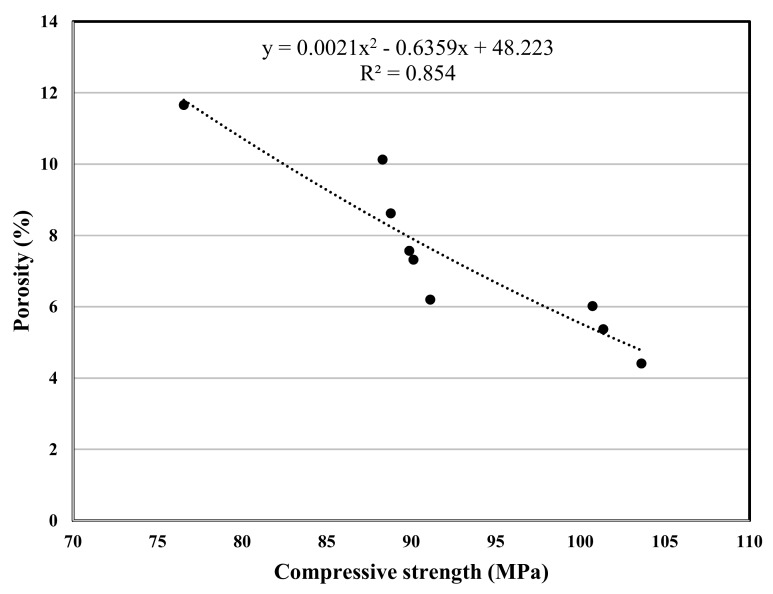
Correlation between compressive strength and porosity.

**Figure 11:**
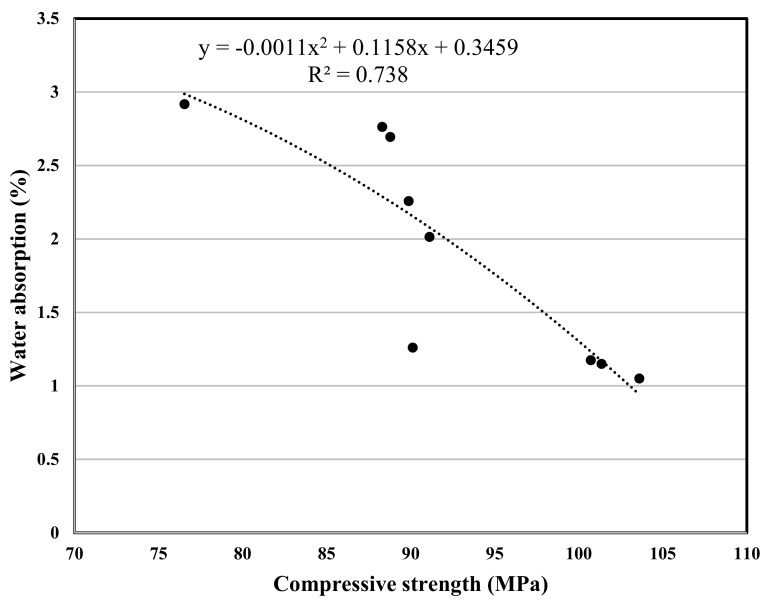
Correlation between compressive strength and water absorption.

**Figure 12:**
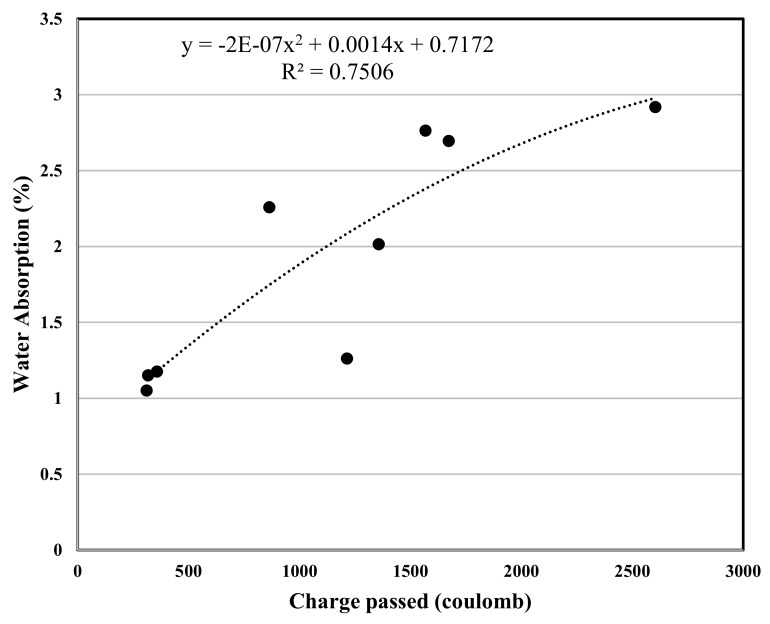
Correlation between charge passing and water absorption.

The experimental program in this study included various concrete tests, the test results are used for a polynomial regression between various properties of concrete, and the related correlation was developed, Figure 7, Figure 8, Figure 9, Figure 10, Figure 11 and Figure 12 show the plot of test results and the related correlation established. It has been developed that the compressive strength of concrete is reduced with the increase in porosity, water absorption, and chloride permeability and vice versa. Therefore, various correlations were established between different test results. Figure 7 shows a strong correlation obtained between the results of compressive strength and the rapid chloride permeability test (the R^2^ was obtained as 0.93). Such correlation obtained in this study is found better than that reported in previous researches [35]. Similarly, Figure 8 and Figure 9 show an excellent correlation between porosity and RPCT (R^2^ is obtained as 0.82) and porosity versus depth of chloride penetration (R^2^ was determined as 0.88). Figure 10, Figure 11 and Figure 12 are showing the correlations between compressive strength versus porosity, compressive strength versus water absorption, and the rapid chloride permeability versus water absorption test results. All correlation established in this research study is found in good agreement with the results of other researchers [43,47].

### 4.4. Statistical Assessment and Optimization of Concrete Containing MK and NS

ANOVA technique was used to perform the statistical analysis of experimental data for onwards application in the response surface methods to determine the optimum mix parameters. The outcomes of ANOVA were optimized by widely accepted RSM techniques [23,48]. The significance level was kept at 0.05 for the quantification of the statistical significance of the experimental outcome. The measured parameters of concrete such as compressive strength, porosity, chloride permeability, and chloride penetration depth were characterized as dependent factors, whereas, MK and NS indicated independent factors. The analysis results are presented in Table 4. The P-value less than 0.05 classify a parameter as significant. The effects of all parameters on compressive strength and porosity and the effects of MK and NS on the rapid chloride permeability test and chloride penetration depth are significant in that respect as given in Table 4. The contribution percentage of parameters shows the effectiveness of each parameter on the tested properties of concrete. The magnitude of the contribution parameter regarding percentages is also presented in Table 4. Based on the results obtained, it is evident that MK showed a significantly excessive contribution to improving the strength and durability characteristics of concrete. Addition of NS with 100% cement (without metakaolin) did not contribute well as it contributed to improving the properties of concrete together with MK content. However, the interaction of both parameters such as metakaolin and nano-silica was also found useful for improving the properties of concrete, which can also be recognized form the magnitudes of the test results. A mathematical relationship between the factors was formulated from the regression model based on the results. Table 5 shows a higher R- square values for the final equations obtained from the analysis. The general equation for the model is represented by Equation (1).

C_p_ = A_1_(MK) + A_2_(NS) + A_3_(MK^2^) + A_4_(NS^2^) + A_5_(MK × NS) + C (1)

where C_p_ is a property of concrete attained from experimental results, i.e., compressive strength, RCP test, chloride penetration depth and porosity, MK and NS are independent linear parameters, (NS^2^) and (MK^2^) are square parameters, (NS×MK) is the interaction parameter, C is a constant, and A_1_ to A_5_ are the coefficients of equation.

**Figure 13:**
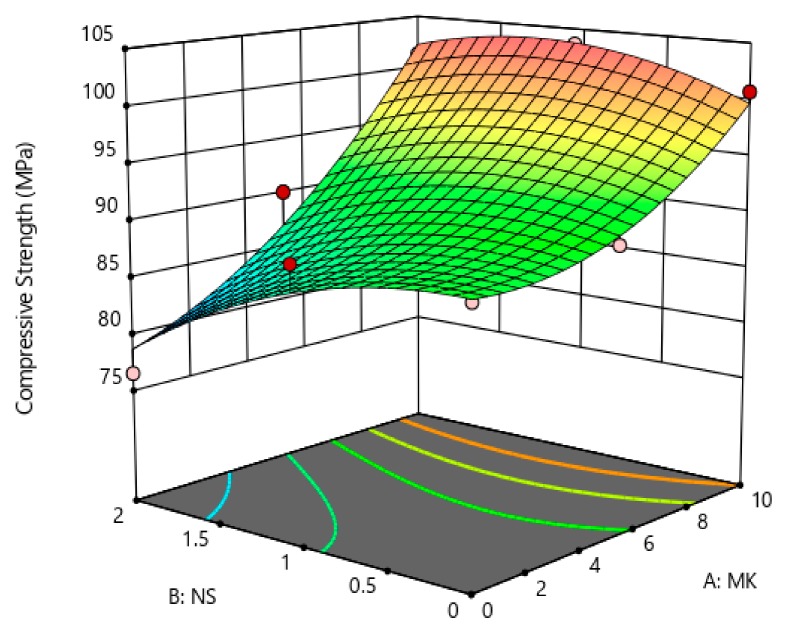
Effect MK and NS proportions on compressive strength.

**Figure 14:**
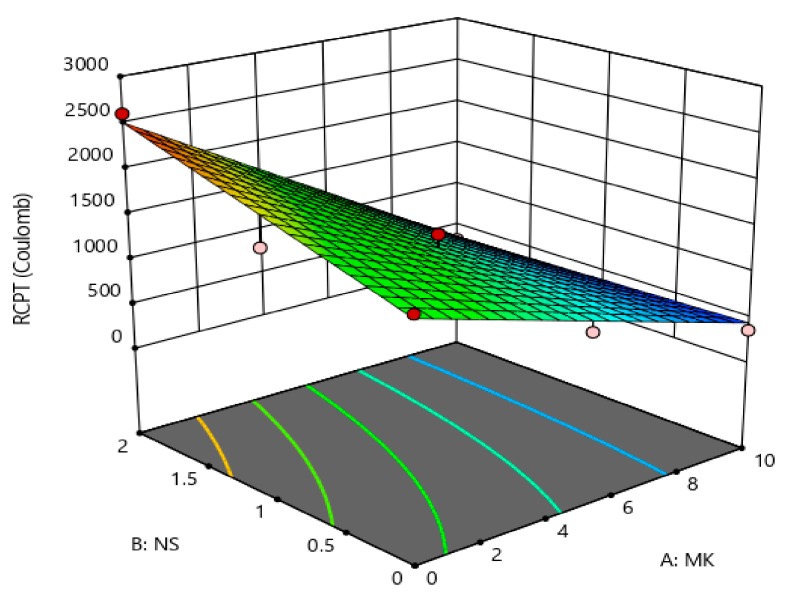
Effect MK and NS proportions on charge passing.

**Figure 15:**
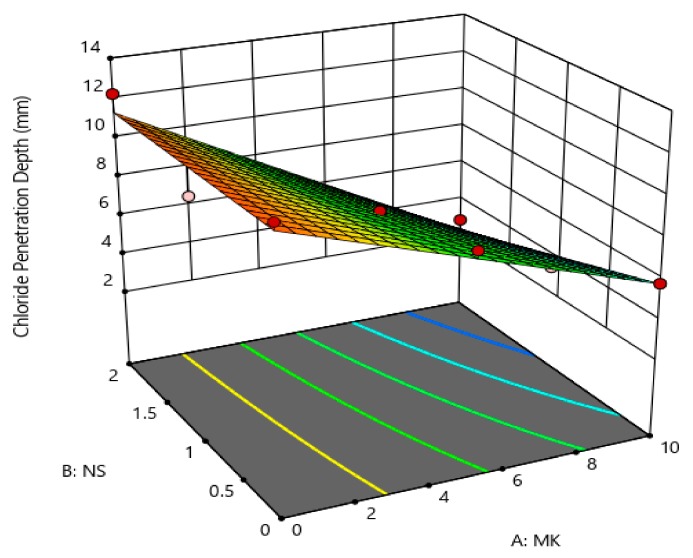
Effect MK and NS proportions on chloride penetration.

**Figure 16:**
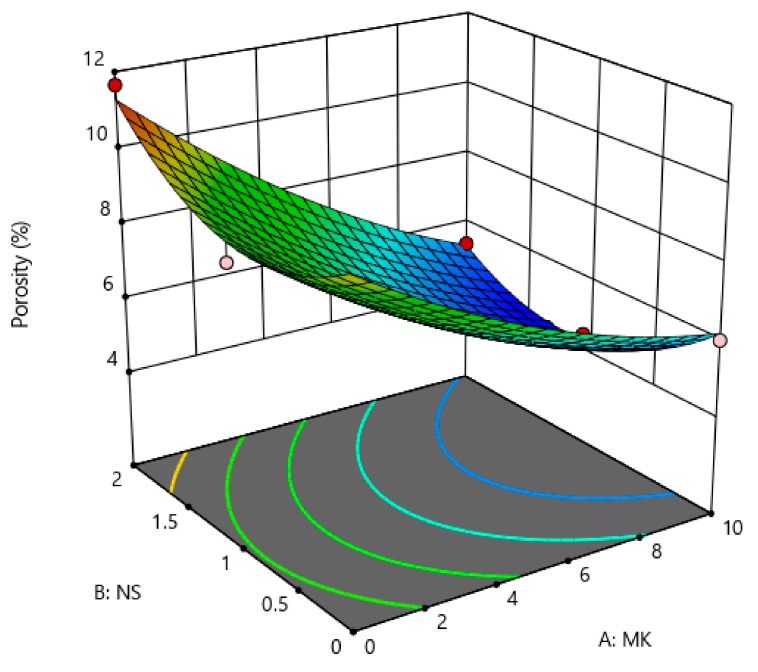
Effect MK and NS proportions on porosity.

The coefficients showed in Table 5 are the part of the empirical models in terms of actual factors (MK and NS) for the compressive strength, rapid chloride permeability, chloride penetration depth, and porosity. The generalized model equation for the responses is represented by Equation (1). Similarly, the final coefficients given in Table 5 can be replaced in Equation (1) to form a modeled equation for each response on tested concrete property. A 3-dimensional (3D) illustration can best describe the relationship between two independent factors (i.e., MK and NS) and the associated responses (i.e., compressive strength, porosity, etc.). Figure 13, Figure 14, Figure 15 and Figure 16 show the 3D response surface plots for CS, RCP, chloride penetration depth, and porosity, respectively. The compressive strength of concrete was enhanced by both MK and NS content in concrete. The increase in MK content the compressive strength increases, whereas, increase the content of nano silica showed a decrease in compressive strength. The relationship between the metakaolin and nano silica content on the compressive strength of concrete is illustrated in Figure 13.

In this research work, the chloride permeability, chloride penetration depth, and porosity were decreased with a higher content of metakaolin and its combination with nano-silica. The relationship between the content of MK and NS and their effects on chloride permeability, chloride penetration depth, and porosity are shown in Figure 14, Figure 15 and Figure 16, respectively.

**Figure 17:**
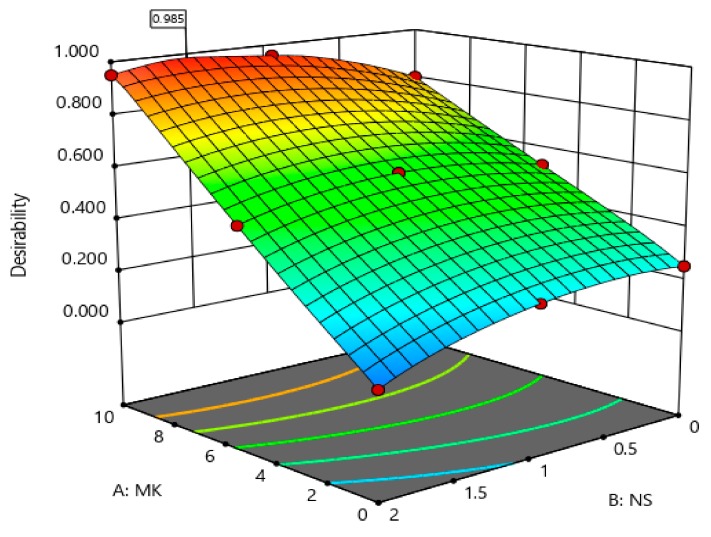
Variation in desirability function based on the multi-objective optimization process on concrete mixes containing MK and NS with cement.

It is not easy to get an optimal solution of all responses simultaneously; therefore, a multi-objective optimization technique was utilized for this study. It is a robust technique for the optimization of all responses at the same time. The optimization was carried out by maximizing the compressive strength while also minimizing the porosity, chloride permeability, and chloride penetration depth. As mentioned, the dependent variables are tested properties of concrete, while two independent variables are proportions of MK and NS. RSM was employed in order to find optimum ratios for maximizing the compressive strength and minimizing the porosity, chloride permeability, and chloride penetration depth. In order to optimize the response, a statistical RSM with multiple responses affected by multiple factors was conducted. The factors and responses are presented in Table 6. Based on the process of multi-objective optimization, three different solutions were obtained, which satisfied the predefined upper and lower range. Desirability represents the closeness of a response to its ideal value. The value closer to one indicates a more desirable solution; in this study, the range of the desirability function varied from 0.948 to 0.985, which specifies the effectiveness of RSM technique. Figure 17 shows the change in desirability function based on the process of multi-objective optimization. The optimum value of 10% MK and 1.55% NS with 0.985 desirabilities for the testing age of 28 days are achieved.

### 4.5. Verification of Data for the Optimum Replacement Ratios

Additional experiments were performed to verify the outcomes obtained from the theoretically obtained response. The primary reason to perform additional experiments is to recognize whether the optimum values could provide minimum chloride permeability, porosity, penetration depth, and the maximum compressive strength by considering the same concrete recipe. The specimen was prepared and tested at the age of 28 days. Table 7 shows the general responses along with experimental responses. Based on the results, it can be concluded that there is significant compatibility between theoretical and experimental responses with a mean error of 2.0%.

## 5. Conclusions

From the results and discussions, the following conclusions have been made: Modified metakaolin (5% to 10%) with 1% or 2% nano-silica content showed a significant enhancement of the mechanical properties of concrete. Increase in compressive strength was observed from 88.3 MPa to 103.6 MPa for the blending proportion of 10% MK with 1% NS, hence, obtained as the best combination. Such combination also caused a significant improvement in the splitting tensile strength (from 5.27 MPa to 6.57 MPa; 25%) and flexural strength (from 5.43 MPa to 7.71 MPa; 42%) as compared to that of the control mix was observed.

The modified metakaolin using nano-silica has tremendously increased the resistance against the penetration of chloride in concrete. The best combination (10% MK + 1%NS) showed a 74% lower value of the chloride penetration depth as compared to the controlled mix. The same combination resulted in the 56% less porosity, a 62% decrease in water absorption, and a 13% reduction in the median pore diameter when compared with the similar performance criteria of the control mix. From the FESEM analysis, the addition of MK and NS revealed the significant enhancement in the microstructure of concrete. 

ANOVA models of the compressive strength, charge passing, chloride penetration depth, and porosity for MK and NS concrete have been developed and statistically validated. All models were significant and ready to use for the prediction of concrete properties.

Based on multi-objective optimization using RSM; the optimum combination of modification was obtained as 10% MK + 1.55% NS. In experimental validation, the optimum combination proved in resulting highest compressive strength and the lowest Porosity, chloride permeability, and chloride penetration depth with the mean error of 2.0%.

## Figures and Tables

**Table 1:** Chemical composition of materials.

Chemical Composition %								Physical Properties	
Constituents	CaO	Al_2_O_3_	SiO_2_	MgO	Fe_2_O_3_	SO_3_	LOI	BET Surface area (m^2^/g)	Density (g/cm^3^)
OPC (Portland cement)	62.85	4.59	25.21	1.70	2.99	−	2	1.066	3.15
MK (metakaolin)	0.58	35.1	53.3	0.27	2.73	0.14	7.4	11.440	2.50

**Table 2:** Physical and chemical composition of nano-silica (NS).

Description of Item	Quality
Appearance	High dispersive white powder
Heat of reduction (%) (105 °C 2 h)	≤3
Loss on ignition (%) (950 °C 2 h)	≤6
SiO2 (dry base) (%)	≥92
SiO2 (dry base) (%) (950 °C 2 h)	≥99.8
Carbon content (%)	≥0.3
Specific surface area (m^2^/g) (BET law)	100±25
PH value	6.5–7.5
Surface density (g/mL)	≤0.15
Dispensability (%) (%) (CCL_4_)	≥80
Oil-absorbed value (mL/100g)	≥250
Average particle size (nm)	10–25

**Table 3:** Mix proportions containing MK and NS.

Mix ID	Factors		Kg for 1 m^3^ Concrete					SP (%)	Slump (mm)	w/b
	MK (%)	NS (%)	Cement	MK	NS	Sand	CA			
CM (100%)	0	0	500	0	0	735	990	0.5	100	0.35
C-MK-5%	5	0	475	25	0	735	990	0.6	101	0.35
C-MK-10%	10	0	450	50	0	735	990	0.83	130	0.35
C-NS-1%	0	1	495	0	5	735	990	0.8	102	0.35
C-NS-2%	0	2	500	0	10	735	990	0.95	107	0.35
C-MK-5% NS-1%	5	1	475	25	5	735	990	1.1	117	0.35
C-MK-5% NS-2%	5	2	475	25	10	735	990	1.35	105	0.35
C-MK-10% NS-1%	10	1	450	50	5	735	990	1.2	130	0.35
C-MK-10% NS-2%	10	2	450	50	10	735	990	1.6	110	0.35

**Table 4:** Analysis of variance results for concrete properties.

Compressive Strength 28 Days	**Source**	**Sum of** **Squares**	**DF**	**Mean** **Square**	**F-Value**	**P-Value**	**Remarks**	**Contribution Parameters**
							
	MK	451.36	1	451.36	133.21	<0.0001	significant	80.15
	NS	19.73	1	19.73	5.82	0.0466	significant	3.50
	MK^2^	32.97	1	32.97	9.73	0.0169	significant	5.85
	NS^2^	20.66	1	20.66	6.10	0.0429	significant	3.67
	MK×NS	38.44	1	38.44	11.35	0.0119	significant	6.83
	Error	0	4	0				
Rapid Chloride Permeability Test 28-Days								
	MK	3933360	1	3933360	111.15	<0.0001	significant	86.93
	NS	301952	1	301952	8.53	0.0170	significant	6.67
	MK×NS	289444	1	289444	8.17	0.0188	significant	6.39
	Error	0	4	0				
Chloride Penetration Depth 28-Days								
	MK	81.77	1	81.77	163.11	<0.0001	significant	90.97
	NS	5.19	1	5.19	10.35	0.0105	significant	5.77
	MK×NS	2.92	1	2.92	5.83	0.0389	significant	3.25
	Error	0	4	0				
							
Porosity 28-Days								
	MK	35.58	1	35.58	364.72	0.0001	significant	80.89
	NS	0.06	1	0.066	0.68	0.4374	-	-
	MK^2^	0.90	1	0.90	9.31	0.0186	significant	2.06
	NS^2^	6.24	1	6.24	63.99	0.0001	significant	14.19
	MK×NS	1.19	1	1.19	12.18	0.010	significant	2.70
	Error	0	4	0				
							

**Table 5:** Coefficients of a mathematical equation of concrete properties.

Equation Coefficients		Concrete Property			
		Compressive Strength (MPa)	Charge Passing (Coulomb)	Chloride Penetration Depth (mm)	Porosity (%)
Constant	C	+91.58	+1207.31	+7.69	6.12
MK	A_1_	+8.67	−809.67	−3.69	−2.44
NS	A_2_	−1.81	+224.33	−0.93	0.10
MK^2^	A_3_	+3.46	-	-	0.57
NS^2^	A_4_	−2.73	-	-	1.50
MK×NS	A_5_	+3.10	−269.0	−0.85	−0.54
R-square		0.95	0.93	0.95	0.98
Adj R-Square		0.93	0.91	0.93	0.97

**Table 6:** Definitions of factors and responses in the multi-objective optimization problem.

Factors and Responses	Goal	Lower Limit	Upper Limit
Metakaolin (%)	In range	0	10
Nano silica (%)	In range	0	2
Compressive strength (MPa)	Maximum	76.54	103.6
RCP (Coulombs)	Minimum	311	2604
Chloride penetration depth (mm)	Minimum	2.8	12.25

**Table 7:** The results of theoretical responses and additional experimental responses of optimal mix design.

Factors and Responses	Optimum Values and Expected Responses	Experimental Results of Optimum Replacement Ratio	Error (%)
MK (%)	10		
NS (%)	1.55		
Compressive strength (MPa)	103.6	104.3	0.67
RCP (coulombs)	375	371	1.33
Chloride penetration depth (mm)	3.14	3.00	4.45
Porosity (%)	4.47	4.40	1.56
Desirability	0.985		
Mean Error			2.00
